# The p53 transcriptional pathway is preserved in ATM^mutated^ and NOTCH1^mutated^ chronic lymphocytic leukemias

**DOI:** 10.18632/oncotarget.2211

**Published:** 2014-07-13

**Authors:** Emmanouil Athanasakis, Elisabetta Melloni, Gian Matteo Rigolin, Chiara Agnoletto, Rebecca Voltan, Diego Vozzi, Elisa Piscianz, Ludovica Segat, Simeone Dal Monego, Antonio Cuneo, Paola Secchiero, Giorgio Zauli

**Affiliations:** ^1^ Institute for Maternal and Child Health, IRCCS “Burlo Garofolo”, Trieste, Italy; ^2^ Department of Morphology, Surgery, Experimental Medicine and LTTA Centre, University of Ferrara, Ferrara, Italy; ^3^ Department of Medical Sciences, University of Ferrara-Arcispedale S. Anna, Ferrara, Italy; ^4^ Cluster in Biomedicine, CBM S.c.r.l., Bioinformatic Services, Area Science Park, Trieste, Italy

**Keywords:** B-CLL, p53, ATM, NOTCH1, Nutlin-3

## Abstract

By using next generation sequencing, we have analyzed 108 B chronic lymphocytic leukemia (B-CLL) patients. Among genes involved in the TP53 pathway, we found frequent mutations in *ATM* (n=18), *TP53* (n=10) and *NOTCH1* (n=10) genes, rare mutations of *NOTCH2* (n=2) and *CDKN1A/p21* (n=1) and no mutations in *BAX*, *MDM2*, *TNFRSF10A* and *TNFRSF10B* genes. The *in vitro* treatment of primary B-CLL cells with the activator of p53 Nutlin-3 induced the transcription of p53 target genes, without significant differences between the B-CLL without mutations and those harboring either *ATM* or *NOTCH1* mutations. On the other hand, the subgroup of *TP53*^mutated^ B-CLL exhibited a significantly lower induction of the p53 target genes in response to Nutlin-3 as compared to the other B-CLL samples. However, among the *TP53*^mutated^ B-CLL, those showing mutations in the high hot spot region of the DNA binding domain [273-280 aa] maintained a significantly higher p53-dependent transcriptional activity as compared to the other *TP53*^mutated^ B-CLL samples. Since the ability to elicit a p53-dependent transcriptional activity *in vitro* has a positive prognostic significance, our data suggest that *ATM*^mutated^, *NOTCH1*^mutated^ and surprisingly, also a subset of *TP53*^mutated^ B-CLL patients might benefit from therapeutic combinations including small molecule activator of the p53 pathway.

## INTRODUCTION

Mutational status of the tumor protein p53 (*TP53*) gene has been one of the most important prognostic molecular markers in B-chronic lymphocytic leukemia (B-CLL) for a long time [1-7], although more recently additional promising novel candidate genes have been described in B-CLL patients as results of whole-exome and/or whole-genome sequencing [8-18]. These technologies provide a novel opportunity to analyze the clonal heterogeneity of the B-CLL genome, with the potential for sensitive detection of mutations restricted to a small fraction of the total tumor cell population. In particular, besides *TP53*, two of the most extensively studied genes in B-CLL involved in p53-pathway are ataxia telangiectasia mutated (*ATM*) [8-11] and *NOTCH1* [12-18]. *ATM* mutation has been reported in up to 16% of B-CLL patients and may be particularly relevant in the setting of del11q, which invariably results in the deletion of one *ATM* allele. Anyhow, sole 11q deletion does not result in *ATM* inactivation by contrast to biallelic defects involving mutations. Interestingly, it has been shown that ATM dysfunction leads to impaired CDKN1A/p21 induction after doxorubicin exposure [11]. Mutations in *NOTCH1* have been described in 8-12% of newly diagnosed B-CLL patients with increasing frequencies (>20%) in advanced disease stages [12-18]. Patients with *NOTCH1* mutations have shorter time to treatment (TTT) and overall survival (OS) independent of other prognostic factors. In this contest, we have previously demonstrated that *in vitro* exposure to the small molecule non-genotoxic activator of the p53 pathway Nutlin-3 activates the NOTCH1 pathway in B-CLL [19]. This finding envisions a negative feed-back loop between p53 and NOTCH1 [19], because a potential mechanism of action of NOTCH1 as oncogene is the suppression of p53-mediated apoptosis through the regulation of p53 stability [20].

Nutlin-3 is a small molecule able to specifically target the p53/MDM2 interaction, leading to the increment of p53 protein levels, transcriptional activation of the p53 molecular targets and, subsequently, to the promotion of cell-cycle arrest and apoptosis induction in a variety of tumor cells [21-24]. Of note, Nutlin-3 and its derivatives have therapeutic perspectives in hematological malignancies [25].

On these bases, a cohort of B-CLL patient samples (n=108) was characterized for the presence of mutations in *TP53* and in a subset of genes related to the p53-pathway, and then analyzed for the transcriptional response to the *in vitro* Nutlin-3 treatment, with particular attention to the induction of CDKN1A/p21, which accurately predict the therapeutic response in B-CLL [26].

## RESULTS

### Targeted deep sequencing analysis of B-CLL

By using a next generation sequencing (NGS) approach, we have performed a mutation screening for *TP53* and for a subset of *TP53* related genes on peripheral CD19^+^ cells obtained from 108 B-CLL patients. As summarized in Table 1, the study population included patients at different disease stage and characterized by different clinical prognostic markers. A total of 216 libraries (2 libraries per B-CLL sample) were sequenced on Ion Chips 316 and 318; quality control and reads alignment to the reference genomic target regions reported an average reads aligned on target at 90.2% with a average depth over 600-fold per sample. Overall, a total of 44 variants were detected in five of the targeted genes (*ATM, CDKN1A, NOTCH1, NOTCH2, TP53*) and were validated in 38 patients (Table 2). These included 31 nucleotide changes already reported in the COSMIC v68 database.

**Table 1:** Characteristics of the whole B-CLL patient study group and of the patient subgroups harboring *TP53*, *ATM* or *NOTCH1* mutations

Characteristics	All patients (n=108)*	*TP53* mutated (n=10)*	*ATM* mutated (n=18)*	*NOTCH1* mutated(n=10)*
Age >70 y	55.5	60	72.2	60
Male	63	70	55.6	70
Rai Stage 0	66	50	72.2	60
Rai Stage I	12.6	0	0	10
Rai Stage II	11.7	10	22.2	20
Rai Stage III	1	0	0	0
Rai Stage IV	8.7	40	5.6	10
IGHV mutated	69.3	60	55.6	60
CD38 high	19.4	10	27.8	50
In therapy	36.1	70	38.9	50
Trisomy 12	15.6	10	11.1	40
13q deletion	63.5	70	100	30
11q deletion	6.3	10	22.2	0
17p deletion	9.4	70	11.1	0
*TP53* mutations	9.3	-	11.1	0
*ATM* mutations	16.7	20	-	10
*NOTCH1* mutations	9.3	0	5.6	-

* all values are expressed as percentage.

**Table 2:** List of NGS mutation validated by Sanger sequencing

Exon	nt change	aa change	COSMIC reported	HLT tumor	Mutated Patients (n)	Freq. %
*ATM*						
8	c.946T>C	p.Tyr316His	No	-	1	51.2
9	c.1229T>C	p.Val410Ala	Yes	Yes, and others	2	50.6, 51.1
12	c.1810C>T	p.Pro604Ser	Yes	Yes	1	48.6
20	c.2932T>C	p.Ser978Pro	Yes	Yes	1	51.2
22	c.3161C>G	p.Pro1054Arg	Yes	Yes	6	43.2 - 46.8
29	c.4388T>G	p.Phe1463Cys	No	-	1	50.0
37	c.5558A>T	p.Asp1853Val	Yes	Yes	2	36.2, 46.8
39	c.5818G>T	Glu1940*	No	-	1	87.8
40	c.5975_5979del	p.Ser1993Argfs*23	No	-	1	55.0
50	c.7342G>A	p.Asp2448Asn	Yes	Yes	1	93.6
50	c.7390T>C	p.Cys2464Arg	Yes	Yes	1	49.7
52	c.7671_7674del	p.Phe2558Leufs*5	No	-	1	89.4
58	c.8492T>C	p.Phe2831Ser	No	-	1	20.9
*CDKN1A*						
2	c.350G>A	p.Cys117Tyr	No	-	1	47.5
*NOTCH1*						
17	c.2734C>T	p.Arg912Trp	No	-	1	50.9
19	c.3011C>T	p.Ser1004Leu	No	-	1	48.5
23	c.3853G>A	p.Val1285Met	No	-	1	54.5
31	c.5690C>T	p.Thr1897Met	No	-	1	48.4
34	c.6941T>C	p.Leu2314Pro	No	-	1	53.5
34	c.7375C>T	p.Gln2459*	Yes	Yes	1	53.2
34	c.7541_7542del	p.Pro2514Argfs*4	Yes	Yes, and others	4	36.1 - 52.8
*NOTCH2*						
22	c.3625T>G	p.Phe1209Val	Yes	Yes	1	46.4
34	c.7242C>G	p.Tyr2414*	No	-	1	44.7
*TP53*						
6	c.376-2A>G	p.Tyr126_Lys132del	Yes	Others	1	94.5
5	c.470T>A	p.Val157Asp	Yes	Others	1	96.2
7	c.701A>G	p.Tyr234Cys	Yes	Yes, and others	1	26.1
7	c.733G>A	p.Gly245Ser	Yes	Yes, and others	1	77.0
7	c.742C>T	p.Arg248Trp	Yes	Yes, and others	1	97.5
7	c.770T>C	p.Leu257Pro	Yes	Yes, and others	1	75.7
8	c.817C>T	p.Arg273Cys	Yes	Yes, and others	2	5.4, 13.4
8	c.818G>A	p.Arg273His	Yes	Yes, and others	1	95.4
8	c.832C>T	p.Pro278Ser	Yes	Others	1	18.9
8	c.838A>G	p.Arg280Gly	Yes	Yes, and others	1	82.3

nt: nucleotide; aa: aminoacid; HLT: hematopoietic and lymphoid tissue; Others: mutation identified in other tumors; Freq.: mutated allele frequency estimated by NGS (multiple values are referred either to 2 different patients or to the range when the mutation was documented in >2 patients).

**Figure 1:**
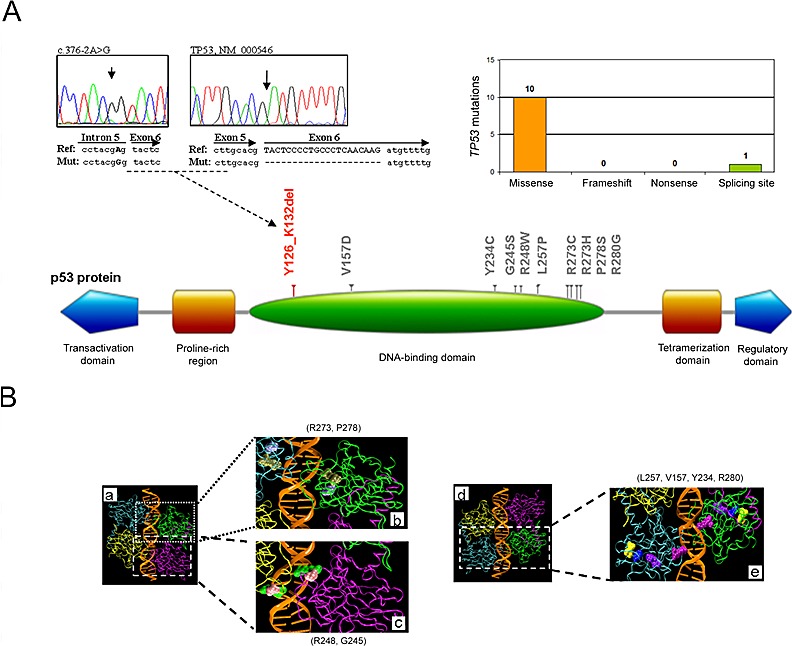
Molecular profile of the TP53 mutations In A, distribution of the *TP53* mutations per type of mutation and schematic representation of the p53 protein domains with indication of the aminoacid alterations: in gray, the non-synonymous mutations; in red, the inframe deletions. Partial electropherograms of the validated splicing site mutation c.376-2A>G on DNA and cDNA, leading to an inframe 7 aminoacid deletion (p.Tyr126_Lys132delYSPALNK) are shown. In B, mutated aminoacids are also evidenced on the structure reconstruction of the p53 tetramer-DNA complex (a and d; PDB accession code 2AC0): (b) tan-R273 and ice blue-P278; (c) green-R248 and pink-G245; (e) yellow-L257, blue-V157, violet-Y234 and purple-R280.

Potentially *TP53* pathogenetic mutations were identified in 10 out of 108 B-CLL patients (9.3%), consistently with previously reported studies carried out with the same genetic approach [27]. These mutations included 10 non-synonymous and 1 splicing site mutation leading to in frame protein deletion of 7 aminoacids (Figure 1A), with one patient harboring 2 different mutations. Of interest, all aminoacid changes afflicted the DNA-binding domain (102-292 aa; Figure 1A-B), with a particular high hot spot of mutations on the protein region involved in the DNA interaction (273-280 aa) [28,29].

**Figure 2:**
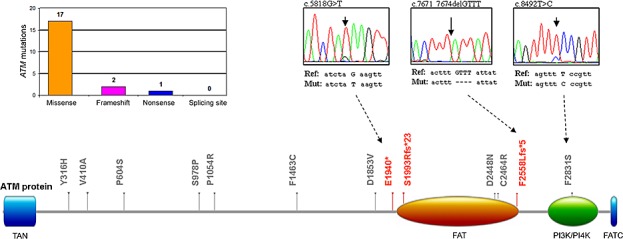
Molecular profile of the ATM mutations Distribution of the *ATM* mutations per type of mutation and schematic representation of the ATM protein domains with indication of the aminoacid alterations: in gray, non-synonymous mutations; in red, nonsense and frameshift deletions. Partial electropherograms of one nonsense (c.5818G>T, p.Glu1940*), one frameshift deletion (c.7471_7674delGTTT, p.Phe2558Leufs*5), and one non-synonymous (c.8492T>C, p.Phe2831Ser) mutations are shown.

**Figure 3:**
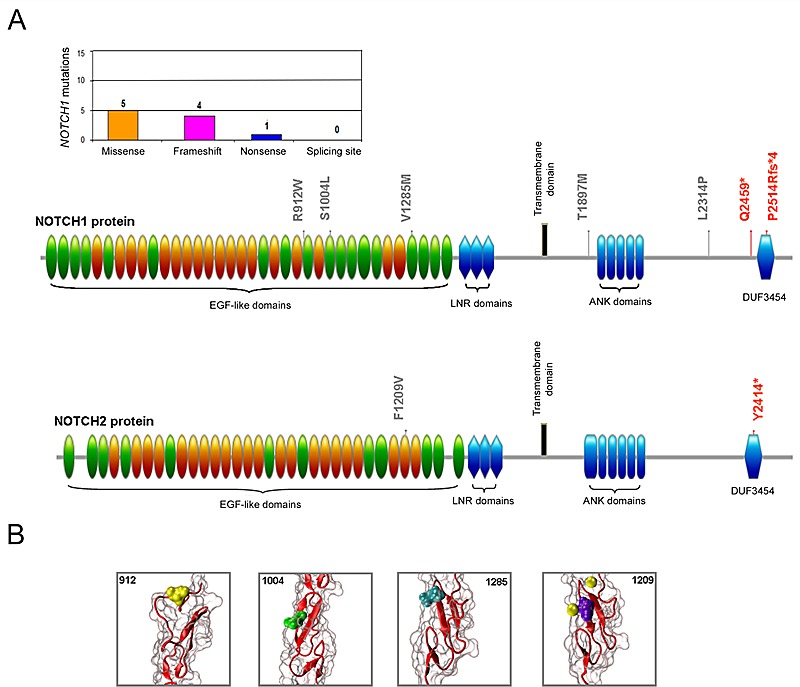
Molecular profile of the NOTCH mutations In A, distribution of the *NOTCH* mutations per type of mutation and schematic representation of the NOTCH1 and NOTCH2 protein domains with indication of the aminoacid alterations. In gray, non-synonymous mutations; in red, nonsense and frameshift deletion mutations. In B, mutated aminoacids are also evidenced on the three-dimensional structure reconstruction of the NOTCH EGF-like domain (PDB accession code 1TOZ). In yellow, cyan and green the mutated aminoacids (positions 912, 1004, and 1285 respectively) identified in NOTCH1 protein. In violet the mutated aminoacid (position 1209) identified in NOTCH2 protein and in yellow the calcium ion that is predicted to bind the wild type aminoacid in position 1209.

In parallel, the analysis of the TP53 functional modulators, such as *ATM, MDM2*, *NOTCH1* and *NOTCH2*, revealed absence of mutations in *MDM2*, while *ATM* mutations were identified in 18 (16.7%) patients and *NOTCH1* and *NOTCH2* mutations were documented in 10 (9.3%) and 2 (1.9%) of the B-CLL patients, respectively (Table 2). In 2 patients *ATM* mutations were coupled to *TP53* mutations (Table 1), and in an additional patient *ATM* mutation was coupled to *NOTCH1* mutation. Studying the potential effect of the 20 identified mutations in the *ATM* gene at protein level, we observed that the point aminoacid changes and deleterious mutation were distributed across the full length of the serine-protein kinase ATM, with 6 mutations affecting the principal functional domains FAT (1960-2566 aa) and PI3K/PI4K (2712-2962 aa) (Figure 2). With respect to NOTCH proteins, among the aminoacid substitutions some were located in the EGF-like domains (24, 26 and 33 for NOTCH1 and 31 for NOTCH2) (Figure 3). On the other side, mutations affecting the protein length (stop codons and frameshift deletions) were located in the intracellular portion, affecting the DUF3454 domain (Figure 3), as previously described [13]. Interestingly, while 20% of *TP53* mutated B-CLL patients also showed *ATM* mutations, none of the patients showed mutations in both *TP53* and *NOTCH1* (Table 1).

Analysis of key p53 transcriptional target genes, such as *CDKN1A*, *BAX* and *TNFRSF10A* and *TNFRSF10B* [21,30,31], revealed mutation of *CDKN1A* in only 1 B-CLL patient (0.9%) (Tables 1 and 2), while no mutations were detected in *BAX*, as well as of genes encoding death receptors *TNFRSF10A* and *TNFRSF10B*.

### Analysis of the transcriptional response of B-CLL to the *in vitro* treatment with Nutlin-3

**Figure 4:**
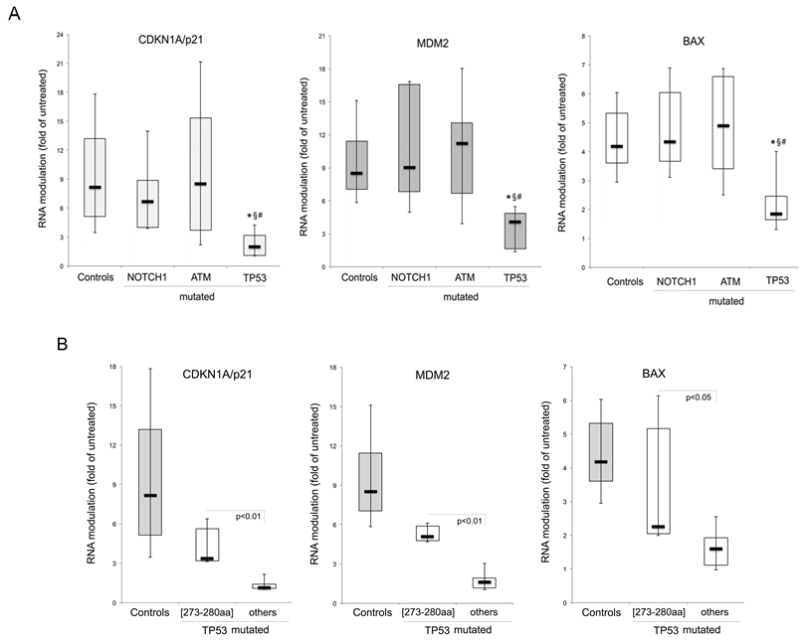
In vitro activation of p53 pathway by Nutlin-3 B-CLL patients' cell cultures were exposed *in vitro* to Nultin-3 (10 microM) for 24 hours before RNA extraction. Transcriptional activation of p53 target genes, CDKN1A/p21, MDM2 and BAX, was assessed by quantitative RT-PCR and mRNA levels were expressed as folds of modulation with respect to the control untreated cultures set at 1. In A, the transcriptional response to Nutlin-3 is compared among the B-CLL samples harboring the indicated mutated genes and those without the investigated mutations (Controls). *, p<0.01 compared to Controls for all target genes; §, p<0.05 (for CDK1A/p21) or p<0.01 (for MDM2 and BAX) compared to NOTCH1^mutated^ samples; #, p<0.05 (for CDK1A/p21) or p<0.01 (for MDM2 and BAX) compared to ATM^mutated^ samples. In B, the transcriptional response to Nutlin-3 is compared between the B-CLL samples harboring *TP53* mutation in different gene loci, as detailed in the text. In A and B, horizontal bars are median, upper and lower edges of box are 75th and 25th percentiles, lines extending from box are 10th and 90th percentiles.

In order to investigate the potential impact of the identified mutations on the functionality of p53 pathway, B-CLL patient derived cell cultures were treated *in vitro* for 24 hours with 10 microM of Nutlin-3, as previously described [19], before assessing the transcriptional activation of canonical target genes. For this purpose, the mRNA levels of CDKN1A/p21, MDM2 and BAX were comparatively measured by quantitative RT-PCR in the untreated and Nutlin-3-treated cultures derived from each B-CLL patient. As shown in Figure 4A, the induction of CDKN1A/p21, MDM2, BAX was comparable among the control group of B-CLL samples, which did not harbor any of the analyzed mutations, and the B-CLL samples characterized by either *ATM* mutations (n=18) or *NOTCH1* mutations (n=10). On the other hand, as expected, the groups of B-CLL samples with *TP53* mutations (n=10) showed a significantly (p<0.01) decreased transcriptional activity as compared to all other groups (Figure 4A). However, it is noteworthy that the subgroup of *TP53* mutated B-CLL samples characterized by single mutations in the high hot spot region of the DNA binding domain (273-280 aa, n=4) still displayed a residual transcriptional activity in response to Nutlin-3 (Figure 4B). Indeed, in this subgroup of patients we documented induction levels of CDKN1A/p21, BAX and MDM2 significantly higher as compared to all other *TP5*3 mutated samples (n=6), in which the mRNA levels of the target genes were unaffected by Nutlin-3 (mean fold induction <2; Figure 4B).

## DISCUSSION

There are numerous reports documenting that *TP53* mutations, with or without del17p, are associated with a poor outcome during first-line treatment [1-7]. However, patients with 17p-CLL exhibit marked clinical heterogeneity, with some patients experiencing an indolent course with prolonged survival [32]. A recent study published by Rossi et al demonstrated that ultra-deep-NGS significantly improved the detection of *TP53* genetic defects in B-CLL allowing the identification of small *TP53* mutated subclones among patients that would be otherwise considered wild type for the *TP53* gene according to Sanger sequencing [27]. In agreement with the study of Rossi et al [27], we also found a *TP53* mutation frequency around 9-10%, with a prevalence of missense substitutions mapping in the DNA-binding domain of the TP53 protein. We and other groups of investigators have previously demonstrated the potential therapeutic efficacy of Nutlin-3, a small molecule non-genotoxic activator of the p53 pathway, in p53^wild-type^ B-CLL cells [21,22]. In the present study, we documented that the presence of *TP53* mutations predicted impairment of the transcriptional activation of TP53 target genes, as assessed by *in vitro* treatment with Nutlin-3. However, we found that the residual transcriptional activity of TP53 mutations toward CDKN1A/p21 and other TP53 target genes significantly varied among the different *TP53*^mutated^ B-CLL patient cell samples. In fact, mutations affecting the hot spot of the DNA binding domain [273-280 aa] were accompanied by partial impairment of the p53 transcriptional activity while mutations of other hot spots abrogated p53 transcriptional activity, as also reported by other studies [28,29,33]. In line with our results, it has been established the presence of a significant correlation between the residual transactivation function of individual *TP53* alleles and clinical variables in patients with inherited p53 mutations who develop cancer [28]. In addition, it has to be underlined the importance of our experimental approach, based on the use of patient leukemic cell cultures, for the evaluation of the p53 transcriptional activity, which has revealed that the DNA binding itself is not sufficient for activating the p53 target genes in at least some of the *TP53* mutants. Therefore, the transcriptional studies performed in yeasts might not always reflect the conditions of primary B-CLL patients cells [33]. Moreover, it is important to specify that the residual p53 transcriptional activity in our primary B-CLL samples harboring mutations affecting the hot spot region [273-280 aa] could not be ascribed to low frequency of the *TP53* mutation, since it was observed also in patient samples in which the mutation frequency was >90% (Table 2).

Another important aim of our study was to assess whether mutations of genes related to the p53 pathway, such as in particular *NOTCH1* and *ATM* might affect the *in vitro* response to Nutlin-3. Therefore, apart the data on the *TP53* mutated B-CLL, it is noteworthy that Nutlin-3 was able to potently induce the transcriptional activity of p53 in *ATM* mutated B-CLL samples. Although these data were not unexpected since ATM works upstream of p53, it is the confirmation that a therapeutic approach based on non-genotoxic activators of the p53 pathway may be beneficial for B-CLL mutated in *ATM*, which represented the more frequent mutation in our patient populations (16.5%), according also to the data of other authors [8-11]. Even more importantly, we demonstrated that Nutlin-3 potently activates a p53 target gene signature also in *NOTCH1* mutated B-CLL cells. These findings are noteworthy for a number or reasons. First, *NOTCH1* mutations are emerging as an independent prognostic factor in B-CLL [12-18]. Second, we have previously demonstrated that Nutlin-3 upregulated NOTCH1 in B-CLL and we have proposed that NOTCH1 activation might represent a negative feed-back loop [19]. Third, it has been shown that NOTCH1 acts by down-modulating the activity of the p53 pathway [20]. Thus, the ability of Nutlin-3 to activate the p53 pathway in *NOTCH1* mutated B-CLL cells is noteworthy since suggests that non-genotoxic activators of p53 might be therapeutically active also in *NOTCH1* mutated B-CLL patients. This is particularly important since novel therapeutic compounds are under investigation for the therapy of B-CLL [34-37]. An additional finding of our study is that mutations of *BAX* and *MDM2* have not been found in our patient study group. Moreover, at variance to other lymphoid malignancies [38,39], also mutations in the death receptors of TRAIL (TRAIL-R1 and TRAIL-R2), which also represent transcriptional targets of Nutlin-3 [40], have not been found in our study, and *CDKN1A/p21* mutation represents a rare event, being observed in only 1 out of 108 patient samples.

Thus, the major conclusion of our study is that the p53 transcriptional pathway is fully preserved in *ATM* and *NOTCH1* mutated B-CLL and partially preserved also in a specific subset of *TP53* mutated patients. In consideration of the low number of *TP53* mutated B-CLL patient samples examined, further studies on a larger cohort are necessary to ascertain whether Nutlin-3, or other non-genotoxic activator of the p53 pathway, might exhibit therapeutic benefits in a subset of *TP53* mutated BCLL patients. Anyhow, these data strength the interest for potential clinical applications of Nutlin-3 also in light of previous reports suggesting that small-molecule p53 activators could have clinical benefits as chemoprotectants for cancer patients bearing p53-mutant tumors, by protecting normal cells from cytotoxicity and nuclear aberrations caused by conventional cancer therapeutics [41,42].

## METHODS

### B-CLL patients

The study population consisted of 108 B-CLL patients. Peripheral blood samples were collected in heparin-coated tubes from all B-CLL patients following informed consent, in accordance with the Declaration of Helsinki and in agreement with institutional guidelines (University-Hospital of Ferrara). The main demographic and clinical parameters of the patients were abstracted from clinical records. B-CLL samples were also characterized by CD38 surface expression, interphase FISH and IgVH status (Table 1). All patients had been without prior therapy at least for three weeks before blood collection. Peripheral blood mononuclear cells (PBMC) were isolated by gradient centrifugation with lymphocyte cell separation medium (Cedarlane Laboratories, Hornby, ON). T lymphocytes, NK lymphocytes, granulocytes and monocytes were negatively depleted from peripheral blood leucocytes (PBL) with immunomagnetic microbeads (MACS microbeads, Miltenyi Biotech, Auburn, CA) and purity (> 93%) of resulting CD19^+^ B-CLL population was assessed by flow cytometry as previously described [24]. Viability of the cells was analyzed at the end of the purification procedure by Trypan blue dye exclusion as previously described [43].

### Targeted deep sequencing and sequencing data analysis

Next generation sequencing of 9 genes related to the p53 pathway, *ATM*, *BAX*, *CDKN1A*, *MDM2*, *NOTCH1*, *NOTCH2*, *TNFRSF10A*, *TNFRSF10B*, and *TP53* itself (Supplemental Table 1) was performed using Ion Torrent chemistry (Life Technologies, Foster City, CA). For each gene, the coding regions (CCDS), untranslated regions (UTR) 5′ and 3′, and also 50bp exons/introns boundaries were targeted by an Ion AmpliSeq custom panel. Two DNA libraries per sample were prepared and as multi-samples pools were subjected to targeted deep sequencing by an Ion Torrent Personal Genome Machine (IT-PGM) platform. A minimum average coverage per sample was fixed at 500-fold.

Sequencing data were aligned to the hg19 human reference genome and variant calling was performed in accord to the Ion Torrent Suite v4 analysis pipeline (Life Technologies). Single Nucleotides Variations (SNVs) and small insertions and deletions (INDELs) were annotated using ANNOVAR software [44], supplied by the COSMIC v68 database (Catalogue Of Somatic Mutations In Cancer, http://cancer.sanger.ac.uk/cancergenome/projects/cosmic/) and several web-tools for the prediction of the pathogenicity of the variants. Alignment of all selected variants was visually verified with the Integrative Genome Viewer v2.2 [45]. Further details are available in the Supplementary Material.

### Variant prioritization and validation

Nucleotide change were prioritized and assigned in two groups: (1) frameshift deletions and insertions, stop codon gain, stop codon loss and splicing site variants; (2) non-synonymous mutations reported in the COSMIC database or predicted pathogenetic by the web-tools (Supplemental Table 2). All selected variants were confirmed either on genomic DNA or, in case of splicing site mutations, on cDNA by Sanger sequencing. Further details and primer sequencers are available in the Supplementary Material.

### Structural bioinformatics analysis

The potential effect of the validated mutations on the protein products was analyzed in terms of sequence comparisons and structural analysis. Protein sequences and structures were retrieved from different databases: NCBI ( http://www.ncbi.nlm.nih.gov/protein/), PDB ( http://pdb.org), UniProt ( http://www.uniprot.org), and PFAM ( http://pfam.sanger.ac.uk). Protein structures were visualized and graphics represented with VMD ( http://www.ks.uiuc.edu/Research/vmd/) and Prosite ( http://prosite.expasy.org/cgi-bin/prosite/mydomains/).

### B-CLL cultures

In order to evaluate the transcriptional functionality of p53, CD19^+^ B-CLL patient cells were seeded at a density of 1×10^6^ cells/ml in RPMI-1640 medium containing 10% FBS, L-glutamine and Penicillin/streptomycin (all from Gibco, Grand Island, NY). Cultures were then either left untreated or exposed to Nutlin-3 (used at 10 microM; Cayman Chemical, Ann Arbor, MI) for 24 hours, before total RNA extraction (QIAGEN RNeasy Plus mini kit; QIAGEN) according to the supplier's instructions. For the integrity measurement, total RNA were analyzed on an Agilent Cary 60 UV-Vis Spectrophotometer (Agilent Technologies Inc., Santa Clara, CA, USA). The expression of relevant p53 target genes (*MDM2, CDKN1A/p21, BAX*) was quantitatively assessed by reverse transcription-polymerase chain reaction (RT-PCR). For this purpose total RNA was transcribed into cDNA, using the QuantiTect^®^ Reverse Transcription kit (QIAGEN). *MDM2, CDKN1A/p21, BAX* gene expression was analyzed using the SYBR Green-based real-time quantitative polymerase chain reaction (RT qPCR) detection method with SABiosciences RT2 Real-TimeTM Gene expression assays, which include specific validated primer sets and PCR master mix (SABiosciences). All samples were run in triplicate using the real time thermal analyzer Rotor-Gene^TM^ 6000 (Corbett, Cambridge, UK), as previously described [23,24]. Expression values were normalized to the housekeeping gene *POLR2A* amplified in the same sample.

## SUPPLEMENTARY MATERIALS AND METHODS TABLES


