# Supplementary material for: The p53 transcriptional pathway is preserved in ATMmutated and NOTCH1mutated chronic lymphocytic leukemias
Source: Oncotarget. 2014 Jul 13;5(24):12635–45. doi: 10.18632/oncotarget.2211 (PMC4350355; doi:10.18632/oncotarget.2211)
Supplement: Supplementary file 1 [file oncotarget-05-12635-s001.pdf]

The p53 transcriptional pathway is preserved in ATM^{mutated} and NOTCH1^{mutated} chronic lymphocytic leukemias

Supplementary Materials and Methods

Nucleic acids extraction and target sequencing design

Genomic DNA was extracted from 5x10^{6} purified B cells by the QIAamp DNA kit (Qiagen, Hilden, Germany). Total RNA was extracted from 5x10^{6} patient-derived B-CLL primary cells using the RNeasy kit (Qiagen). DNA and RNA quantity and quality was assessed using Nanodrop ND-1000 spectrophotometer and agarose gel electrophoresis.

The UCSC Genome Browser (http://genome.ucsc.edu/) was used to select the genomic coordinates of the exonic sequences of *ATM*, *BAX*, *CDKN1A*, *MDM2*, *NOTCH1*, *NOTCH2*, *TNFRSF10A*, *TNFRSF10B*, and *TP53* genes. A primer pool, intended for DNA library construction through multiplex PCR, was defined by Ion AmpliSeq™ Designer v1.2.6 (Life Technologies, Foster City, CA). The primers design ensures 86.0% overall targeted region coverage spanning through 58.9Kb; the missing percent of bases correspond at~2.0% in CCDS regions and at ~12.0% in UTR regions. To test our custom panel and to set up the variant calling parameters, we prepared and run on Ion Chip 314 DNA libraries derived from the p53^{mutated} B lymphoblastoid leukemic cell lines MAVER, MEC-1 and MEC-2 (Deutsche Sammlung von Mikroorganismen und Zellkulturen GmbH, Braunschweig, Germany).

Library construction and targeted DNA sequencing

A sample library preparation was performed using 10 ng of purified genomic DNA through Ion AmpliSeq Library Kit v2.0; each library were indexed using Ion Xpress Barcode Adapters Kit (Life Technologies) in accord to the manufacturer's protocols. DNA libraries were purified by AMPure XP magnetic beads (Beckman Coulter, Fullerton, CA) and quantified using Ion Library TQMN Quantification kit (Life Technologies). Template Ion Sphere Particles were arranged using Ion PGM Template OT2 200 kit and a single end 200 base-read sequencing run was carried out on IT-PGM system (Life Technologies). Pool of indexed patient libraries were sequenced on Ion Chips (316, 100Mb output; 318, 1Gb output).

Sequencing data annotation

SNVs and INDELs were then annotated using ANNOVAR software, referring to the following available public databases: RefGene, NCBI dbSNP build137 (http://www.ncbi.nlm.nih.gov/SNP/), 1000 Genomes Project (http://www.1000genomes.org/), NHLBI-ESP (http://evs.gs.washington.edu/EVS/), COSMIC v68 (http://cancer.sanger.ac.uk/cancergenome/projects/cosmic/), SIFT (http://sift.jcvi.org/), PolyPhen-2 (http://genetics.bwh.harvard.edu/pph2/), MutationTaster (http://www.mutationtaster.org/), MutationAssessor (http://mutationassessor.org/), RPOVEAN (http://provean.jcvi.org/seq_submit.php) and SNPEffect (http://snpeffect.switchlab.org/). To detect alterations in exon–intron boundary regions and splicing motifs due to nucleotide changes, the bioinformatic tools for splice site prediction

HSF (http://www.umd.be/HSF) and NNSplice (http://www.fruitfly.org/seq_tools/splice.html) were used.

Variants quality control

Variants were called using the “Somatic - PGM - Low Stringency” configuration of Torrent Variant Caller plugin v4, with a MAF value at 0.02 for the SNVs and at 0.05 for the INDEL. All variants were filtered by quality (QUAL) and coverage (CV), excluding SNVs with a CV value <10 and a QUAL value <20. For the INDELs the cutoff of QUAL value was setting at 30.

Sanger sequencing validation

All selected variants were confirmed by direct sequencing on an ABI 3500Dx Genetic Analyzer (Life Technologies, Foster City, CA, USA) using the BigDye v3.1 Terminator Cycle Sequencing mixture (Life Technologies) according to the manufacturer’s instructions. Primers were designed using Primer3 v4 software (http://primer3.ut.ee/) (Supplementary Table 3) and amplification of the corresponding genomic and cDNA regions was conducted by a general PCR method. In case of the cDNA sequencing, first was performed a reverse transcription reaction with specific primers on mRNA samples in accord to the manufacturer’s protocols of Transcriptor First Strand cDNA Synthesis Kit (Roche, Waiblingen, Germany).

Supplementary Table 1: Selected genes for the Ion AmpliSeq Design.

		NCBI reference ID		
Gene	Description	mRNA	CCDS	Protein
ATM	Serine-protein kinase ATM	NM_000051.3	CCDS31669.1 (9171 nt)	NP_000042.3 (3056 aa)
BAX	Apoptosis regulator BAX	NM_004324.3	CCDS12744.1 (657 nt)	NP_004315.1 (218 aa)
CDKN1A	Cyclin-dependent kinase inhibitor 1 (p21)	NM_000389.4	CCDS4824.1 (495 nt)	NP_000380.1 (164 aa)
MDM2	E3 ubiquitin-protein ligase Mdm2	NM_002392.5	CCDS8986.2 (1494 nt)	NP_002383.2 (497 aa)
NOTCH1	Neurogenic locus notch homolog protein 1	NM_017617.3	CCDS43905.1 (7668 nt)	NP_060087.3 (2555 aa)
NOTCH2	Neurogenic locus notch homolog protein 2	NM_024408.3	CCDS908.1 (7416 nt)	NP_077719.2 (2471 aa)
TNFRSF10A	Tumor necrosis factor receptor superfamily member 10a (TRAIL-R1)	NM_003844.3	CCDS6039.1 (1407 nt)	NP_003835.3 (468 aa)
TNFRSF10B	Tumor necrosis factor receptor superfamily member 10b (TRAIL-R2)	NM_147187.2	CCDS6036.1 (1236 nt)	NP_671716.2 (411 aa)
TP53	Cellular tumor antigen p53	NM_000546.5	CCDS11118.1 (1182 nt)	NP_000537.3 (393 aa)

CCDS: consensus coding sequence.

Supplementary Table 2: Overview of the pathogenetic prediction of all nonsynonymous selected variants.

Variant informations	SIFT|PP2|LRT|MT	MA	PROVEAN	SNPEffect
ATM:NM_000051:exon7:c.G703A:p.A235T	D|D|D|N	Medium	Neutral	-
ATM:NM_000051:exon8:c.T946C:p.Y316H	D|D|D|N	Medium	Deleterious	-
ATM:NM_000051:exon9:c.T1229C:p.V410A	D|P|D|N	Low	Neutral	-
ATM:NM_000051:exon12:c.C1810T:p.P604S	T|B|D|N	Low	Neutral	-
ATM:NM_000051:exon20:c.T2932C:p.S978P	D|D|D|N	medium	Deleterious	-
ATM:NM_000051:exon22:c.C3161G:p.P1054R	D|D|D|N	Medium	Deleterious	-
ATM:NM_000051:exon29:c.T4388G:p.F1463C	D|D|D|D	Medium	Deleterious	-
ATM:NM_000051:exon37:c.A5558T:p.D1853V	D|D|D|D	Medium	Deleterious	-
ATM:NM_000051:exon50:c.G7342A:p.D2448N	T|D|D|D	Medium	Deleterious	-
ATM:NM_000051:exon50:c.T7390C:p.C2464R	T|D|D|N	Low	Deleterious	-
ATM:NM_000051:exon58:c.T8492C:p.F2831S	D|D|D|D	Medium	Deleterious	-
BAX:NM_004324:exon2:c.G86T:p.G29V	D|D|N|NA	Low	Deleterious	-
CDKN1A:NM_000389:exon2:c.G350A:p.C117Y	D|D|D|D	Low	Deleterious	-
MDM2:NM_002392:exon9:c.A836C:p.D279A	D|D|D|NA	Low	Deleterious	-
NOTCH1:NM_017617:exon3:c.A311C:p.N104T	D|B|D|D	Medium	Deleterious	-
NOTCH1:NM_017617:exon11:c.G1772C:p.C591S	D|D|D|D	High	Deleterious	-
NOTCH1:NM_017617:exon17:c.C2734T:p.R912W	D|D|N|D	Neutral	Deleterious	-
NOTCH1:NM_017617:exon19:c.C3011T:p.S1004L	D|P|D|D	Medium	Deleterious	-
NOTCH1:NM_017617:exon21:c.T3445C:p.C1149R	D|D|D|D	High	Deleterious	-
NOTCH1:NM_017617:exon22:c.A3569G:p.H1190R	D|P|D|N	Neutral	Neutral	-
NOTCH1:NM_017617:exon23:c.G3853A:p.V1285M	D|P|D|D	Medium	Neutral	-
NOTCH1:NM_017617:exon31:c.C5690T:p.T1897M	D|D|D|D	Low	Deleterious	-
NOTCH1:NM_017617:exon34:c.G6181C:p.E2061Q	D|P|D|D	Neutral	Deleterious	-
NOTCH1:NM_017617:exon34:c.T6941C:p.L2314P	D|P|D|NA	Low	Deleterious	-
NOTCH2:NM_024408:exon22:c.T3625G:p.F1209V	D|D|U|D	Medium	Deleterious	-
TP53:NM_000546:exon5:c.T470A:p.V157D	D|D|N|D	Medium	Deleterious	RS
TP53:NM_000546:exon7:c.A701G:p.Y234C	D|D|D|D	Medium	Deleterious	RS
TP53:NM_000546:exon7:c.T706G:p.Y236D	D|D|N|D	Medium	Deleterious	SERS
TP53:NM_000546:exon7:c.G733A:p.G245S	D|D|D|D	Medium	Deleterious	RS
TP53:NM_000546:exon7:c.C742T:p.R248W	D|D|D|D	Medium	Deleterious	RS
TP53:NM_000546:exon7:c.T770C:p.L257P	D|D|D|D	Medium	Deleterious	SERS
TP53:NM_000546:exon8:c.C817T:p.R273C	D|D|D|D	Medium	Deleterious	SLRS
TP53:NM_000546:exon8:c.G818A:p.R273H	D|P|D|D	Medium	Deleterious	NES
TP53:NM_000546:exon8:c.G824T:p.C275F	D|D|D|D	Medium	Deleterious	NES
TP53:NM_000546:exon8:c.C832T:p.P278S	D|D|D|D	Medium	Deleterious	SERS
TP53:NM_000546:exon8:c.A838G:p.R280G	D|D|D|D	Medium	Deleterious	RS

SIFT: sorting intolerant from tolerant; PP2: polymorphism phenotyping v2; LRT: likelihood ratio test; MT: mutation taster; MA: mutation assessor impact; PROVEAN: protein variation effect analyzer. D: damage; N: neutral; T: tolerant; B: begnin. SERS: severely reduced stability; SLRS: slightly reduced stability; RS: reduced stability; NES: no effect on stability.

Supplementary Table 3: Sanger sequencing primers targeting the regions of all validated mutations.

Chromosome and start-end genomic position	Forward 5’ to 3’	Reverse 5’ to 3’
ATM		
chr11:108117735-108117735	GCTGATGCAGCTTGACAGCT	GTGACATGACCTACTTACTGT
chr11:108119823-108119823	ATCTTACACTACTACACAAAGAG	TGGCCCAAGCAAATGAGCCA
chr11:108123551-108123551	ATGGTTGTCCTCCTTAAATTGTC	TAAGATGCAGCTACTACCCAGCT
chr11:108141988-108141988	GTTCTTGAACTTCTGAAACCAC	TCCCTTGTGTTCTCAGAGTC
chr11:108143456-108143456	TTGCTTGAGGTGAGTTTTTGC	TACTGCCATCTGCAGCATTC
chr11:108160480-108160480	TGTGAGCAAGCAGCTGAAACA	GACAGAGTGAGTCTTTGTCTC
chr11:108175463-108175463	AATATGTCAACGGGGCATGA	CATTCCATAGATGAAGAAAC
chr11:108180942-108180942	TGTTAAGCAGTCACTACCATTG	TCCATTACCTTTTCTCTTGATC
chr11:108183194-108183198	GTATATGTATTCAGGAGCTTCCA	TGGCAGAGTCAGTATTAGAACTCAAG
chr11:108200975-108200975	GCAAGATACACAGTAAAGGTTCAG	CTTCATCATGCCATTGACTTC
chr11:108201023-108201023	GCAAGATACACAGTAAAGGTTCAG	CTTCATCATGCCATTGACTTC
chr11:108202647-108202650	TACCCACTGCAGTATCTAGAC	TCAAGCTGAGAGCTTTGTTTAG
chr11:108216543-108216543	GCTTCCCTGTCCAGACTGTTAG	CCTGCCAAACAACAAAGTGCTC
CDKN1A		
chr6:36652228-36652228	AGACACCACTGGAGGGTGAC	CCCTTGGACCATGGATTCTGA
NOTCH1		
chr9:139390649-139390650	GATCTGGGACTGCATGCTG	GCGGTGCACACTATTCTGC
chr9:139390816-139390816	GCAGGTGCAGCCACAAAACT	ACAGGCGAGGAGTAGCTGTG
chr9:139391250-139391250	CTTTGAGACTGGCCCACCTC	AGGCCCTGGTAGCTCATCAT
chr9:139395248-139395248	TAAGCTGGATTCGGCTCTGTC	GGCATCAGAGCGTGAGTAGC
chr9:139401216-139401216	GCAGGTGTGCACTGTGAGAT	GTAAGAGCAGGGCAGTGAGA
chr9:139403482-139403482	AGGTGTGACGTGGTGTGAGAG	AGTTGGGGCCAGTGTAGCCCT
chr9:139405111-139405111	ATCAACGAGTGCGTTCTGAG	ATCCTCGGCTCAGTGAAGAG
NOTCH2		
chr1:120478125-120478125	TGCTTGGAATGAAGCTAGTCT	CAGGCTATCAGGGTGTCAAC
chr1:120458103-120458103	CTATCATCCTTTCCCAGCCT	TTGTGTGGTGGCTCAGACATG
TP53		
chr17:7577100-7577100	TTCTTGTCCTGCTTGCTTACC	GCTTCTCCTCCACCTACCT
chr17:7577106-7577106	TTCTTGTCCTGCTTGCTTACC	GCTTCTCCTCCACCTACCT
chr17:7577120-7577120	TTCTTGTCCTGCTTGCTTACC	GCTTCTCCTCCACCTACCT
chr17:7577121-7577121	TTCTTGTCCTGCTTGCTTACC	GCTTCTCCTCCACCTACCT
chr17:7577511-7577511	TAAGAGGTCCCAAAGCCAGA	CTTGGGCCTGTGTTATCTCC
chr17:7577539-7577539	TAAGAGGTCCCAAAGCCAGA	CTTGGGCCTGTGTTATCTCC
chr17:7577548-7577548	TAAGAGGTCCCAAAGCCAGA	CTTGGGCCTGTGTTATCTCC
chr17:7577580-7577580	TAAGAGGTCCCAAAGCCAGA	CTTGGGCCTGTGTTATCTCC
chr17:7578460-7578460	CTCGCTAGTGGGTTGCAGG	TATCTGAGCAGCGCTCATGG
chr17:7578556-7578556	CTCGCTAGTGGGTTGCAGG	TATCTGAGCAGCGCTCATGG
**CCDS ID and start-end cDNA position**	**Forward 5’ to 3’**	**Reverse 5’ to 3’**
TP53		
CCDS11118: 299-566	AGAAAACCTACCAGGGCAGC	GCCAGACCATCGCTATCTGA
CCDS11118: 299-689	AGAAAACCTACCAGGGCAGC	GAGGTTGGCTCTGACTGTAC
